# Inoculation With *Azospirillum brasilense* and *Bacillus amyloliquefaciens* Enhances Tomato Resilience to Severe Water Deficit: A Comprehensive Morpho‐Physiological and Biochemical Analysis

**DOI:** 10.1111/1758-2229.70316

**Published:** 2026-04-28

**Authors:** Camila Garcia de Freitas, Mádilo Lages Vieira Passos, Mariana de Vasconcelos Dias, Leandro Israel da Silva, Lilian Ferreira de Sousa, Filipe Almendagna Rodrigues, Caroline Dambroz, Vytória Piscitelli Cavalcanti, Joyce Dória

**Affiliations:** ^1^ Department of Agriculture Federal University of Lavras (UFLA) Lavras Brazil; ^2^ Department of Water Resources Federal University of Lavras (UFLA) Lavras Brazil; ^3^ Department of Biology Federal University of Lavras (UFLA) Lavras Brazil; ^4^ Department of Entomology Federal University of Lavras (UFLA) Lavras Brazil

**Keywords:** oxidative stress, physiological responses, plant growth‐promoting bacteria, tomato, water deficit

## Abstract

Water scarcity, intensified by climate change, threatens tomato ( *Solanum lycopersicum* ) productivity, impacting growth, metabolism and secondary metabolite production. This study evaluated the effects of *Azospirillum brasilense* and *Bacillus amyloliquefaciens* inoculation in mitigating water stress in the Ibiza cultivar under 100%, 50% and 25% water replenishment. Severe water deficits significantly reduced morphological parameters, such as height, leaf number and dry mass. However, inoculation with *B. amyloliquefaciens* , particularly in combination with *A. brasilense* , enhanced dry mass accumulation, stem diameter and root development. Physiological analyses revealed higher electrolyte leakage and compromised membrane integrity due to water stress. Biochemical responses included increased antioxidant enzyme activity (SOD, CAT and APX) and proline accumulation, both modulated by bacterial inoculation. Interestingly, chlorophyll content increased at 25% water availability, suggesting an adaptive mechanism, while DNA content decreased. Secondary metabolites (flavonoids and phenolics) also responded to the interaction between irrigation levels and bacterial inoculation. The findings demonstrate that inoculation with *A. brasilense* and *B. amyloliquefaciens* is an effective approach to enhance tomato tolerance to water deficits by improving agronomic performance, strengthening antioxidant defences and regulating secondary metabolite production. This strategy supports the development of sustainable and adaptive agricultural practices in the face of increasing water scarcity.

## Graphical abstract

Inoculation with *Azospirillum brasilense* and *Bacillus amyloliquefaciens* significantly enhances tomato tolerance to severe water deficit. This strategy boosts agronomic performance, strengthens antioxidant defences and regulates secondary metabolites, providing a sustainable agricultural approach to combat water scarcity and climate change impacts.

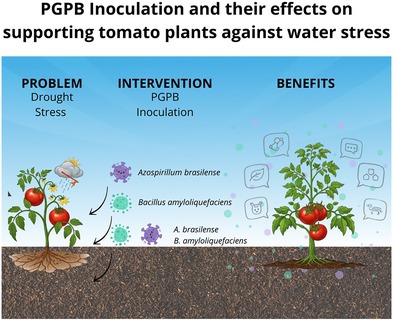

## 1 Introduction

Tomato ( *Solanum lycopersicum* L.) is a globally significant crop because it is used as a fresh food and as a raw material in the industrial production of derivatives (Rivero‐Pino 2023). However, its productivity can be threatened by abiotic factors such as water deficit, which has intensified due to the ongoing climate change impacting major production regions (Di Laccio et al. 2024).

Water stress compromises plant growth, reduces photosynthetic efficiency, negatively affects metabolism and consequently leads to significant loss of productivity (Tripodi et al. 2025). In addition to agronomic implications, water stress alters the production of secondary metabolites that are essential for plant defence and the nutritional quality of fruits, including flavonoids and carotenoids (Ru et al. 2023).

In this context, the use of agricultural strategies to mitigate the effects of water stress is critical for achieving efficient crop management and maintaining high productivity. With the growing demand for high‐quality, nutritionally rich food cultivated in more sustainable production systems, the adoption of biotechnological practices, such as the inoculation of plant growth‐promoting bacteria (PGPB), has emerged as a promising strategy (Kang et al. 2025).

These bacteria act as biostimulants or phyto‐stimulators by regulating the synthesis of phytohormones, such as auxins (IAA), cytokinins and gibberellins, and by triggering physiological responses. This leads to altered plant metabolism, accelerated growth, greater physiological plasticity and improved adaptation to different environmental conditions (Lopes et al. 2021; Shahid et al. 2023; Nascimento et al. 2021).

Microbial‐based bioinputs, including *Azospirillum brasilense* and *Bacillus amyloliquefaciens* , are notable for their ability to promote root growth, enhance soil exploration by the roots and improve water uptake efficiency. They also positively influence plant physiology under water‐stress conditions (Kang et al. 2025; Zarea 2025).

Thus, the present study aimed to evaluate the effects of *A. brasilense* and *B. amyloliquefaciens* inoculation on mitigating water stress in *S. lycopersicum* , with emphasis on agronomic performance, stress tolerance and the impact on secondary metabolite production. This approach seeks to contribute to the development of more sustainable and adaptable agricultural practices to address increasing water limitations while also offering new insights into the benefits of PGPB use for improving the quality and functionality of agricultural production.

## 2 Materials and Methods

### 2.1 Production of *Solanum lycopersicum* Seedlings

The seedlings of *S. lycopersicum* (cv Ibiza) were grown in a greenhouse using polyethylene trays filled with commercial substrate (SV vegetal substrate, composed of peat and pine bark), with two seeds per cell. After 30 days, the seedlings were transplanted into 3 L plastic pots. The pots had drainage holes at the base and a gravel layer to facilitate drainage. The remaining volume of the pots was filled with a mixture of soil and commercial substrate at a 2:1 ratio.

Based on chemical analysis, the soil used had the following composition: pH: 5.3; K: 94.00 mg/kg (94.00 mg/dm^3^); P: 1.54 mg/kg (1.54 mg/dm^3^); Ca: 52.01 mg/kg (2.60 cmolc/dm^3^); Mg: 8.87 mg/kg (0.73 cmolc/dm^3^); Al: 0.04 cmolc/dm^3^; H + Al: 4.10 cmolc/dm^3^; organic matter: 4.7%; Zn: 3.90 mg/kg (3.90 mg/dm^3^); Fe: 43.40 mg/kg (43.40 mg/dm^3^); Mn: 91.10 mg/kg (91.10 mg/dm^3^); Cu: 8.0 mg/kg (8.0 mg/dm^3^); B: 0.65 mg/kg (0.65 mg/dm^3^) and S: 0.87 mg/kg (0.87 mg/dm^3^). The soil was classified as clay.

### 2.2 Microorganisms, Inoculum Standardisation and Inoculation Procedures

In this study, *A. brasilense* (CCMA 1291) and *B. amyloliquefaciens* (CCMA 0112) were selected from the Culture Collection of Agricultural Microbiology (CCMA/UFLA). Primary solid nutrient agar medium (comprising bacteriological peptone, yeast extract, NaCl and agar) in Petri dishes was maintained under controlled conditions at 25°C for 48 h. The bacterial inoculum was prepared by resuspending the bacterial biomass collected using a sterile inoculation loop in autoclaved water. The inoculum was standardised by turbidity (0.5 McFarland Standard), achieving a final concentration of 1.5 × 10^8^ CFU·mL^−1^ (Lashani et al. 2023).

Inoculation was performed separately for *A. brasilense* and *B. amyloliquefaciens* , as well as in combination with both. It was applied three times, at an interval of 21 days between each application. For individual treatments, 10 mL of inoculum from each bacterium was applied directly to the soil near the plant roots. In the combined treatment, a mixture of 5 mL *A. brasilense* inoculum and 5 mL *B. amyloliquefaciens* inoculum was applied (totalling 10 mL per plant), maintaining the final bacterial cell concentration.

### 2.3 Irrigation Levels and Experimental Design

In addition to the bacterial treatments described in the previous section, three irrigation levels were applied, corresponding to 100%, 50% and 25% water replacement (Table 1). Furthermore, a control treatment without bacterial inoculation was included for all the irrigation levels.

**TABLE 1:** The treatments were based on a combination of different inoculants ( *Azospirillum brasilense* , *Bacillus amyloliquefaciens* and a solution of *A. brasilense* + *B. amyloliquefaciens* ) with different irrigation levels (100%, 50% and 25% water replacement).

Treatments	Composition
L1T1	25% water replenishment line with non‐inoculated plants
L1T2	25% water replenishment line with plants inoculated with *A. brasilense*
L1T3	25% water replenishment line with plants inoculated with *B. amyloliquefaciens*
L1T4	25% water replenishment line with plants inoculated with *A. brasilense* and *B. amyloliquefaciens*
L2T1	50% water replenishment line with non‐inoculated plants
L2T2	50% water replenishment line with plants inoculated with *A. brasilense*
L2T3	50% water replenishment line with plants inoculated with *B. amyloliquefaciens*
L2T4	50% water replenishment line with plants inoculated with *A. brasilense* and *B. amyloliquefaciens*
L3T1	100% water replenishment line with non‐inoculated plants
L3T2	100% water replenishment line with plants inoculated with *A. brasilense*
L3T3	100% water replenishment line with plants inoculated with *B. amyloliquefaciens*
L3T4	100% water replenishment line with plants inoculated with *A. brasilense* and *B. amyloliquefaciens*

A randomised complete block design (RCBD) in a split‐plot arrangement was adopted, with two factors: three irrigation levels (main plots) and three bacterial inoculants + control (subplots), resulting in 12 treatments. Each treatment consisted of five replicates, with two plants per replicate. For each variable of interest, the value of a replicate corresponded to the mean value of the two plants, resulting in five experimental units per treatment and a total of 120 plants in the experiment.

To ensure proper randomisation within the blocks and facilitate irrigation delivery, the experimental area within the greenhouse was divided into five spatial blocks. Within each block, all three irrigation levels (100%, 50% and 25% water replacement) were randomly assigned to the main plot. Each main plot consisted of a section of the irrigation system designed to deliver the specific water volume required, ensuring that the main plots were randomly distributed across the block, thereby mitigating potential environmental gradients. Subsequently, within each main plot, the four bacterial treatments were randomly assigned to individual plant subplots (Table S1).

The irrigation hoses used had a nominal diameter of 16 mm and thin walls. The three lateral irrigation lines were designed to allow differentiated water delivery to the randomised main plots within each block. Each line was equipped with 10 drippers and 40 drip stakes, totalling 40 pots per lateral line. This physical setup supported the application of the distinct irrigation levels to their respective, randomly allocated main plots across the blocks.

To determine the pot weight at field capacity and establish the defined irrigation levels (100%, 50% and 25%), four pots from the treatment corresponding to 100% irrigation were selected for analysis. The pots were saturated using the capillarity method (Casaroli and van Jong Lier 2008), in which the pots were submerged in a tank filled with water up to two‐thirds of their height. During the saturation process, the pots were submerged for 24 h, with the surface covered with a plastic film to prevent evaporation.

After the saturation period, the pots were removed from the water tank and placed on a bench inside the greenhouse, where they were left to rest and allow free drainage of excess water. This process continued until the weight of the pot substrate stabilised, representing the field capacity. During the drainage process, the pots were weighed three times a day (at 8 AM, 12 PM and 4 PM) at fixed times. Stabilisation was achieved when weight variations between consecutive days were negligible.

After 3 days of drainage, a constant weight was achieved, thereby determining the field capacity of the pots. Once the weight of the pots at field capacity was established, a drip irrigation system was adopted. This system used online emitters (CLICKTIF HD PC 2 L h^−1^ model) with a pressure regulation range of 0.50–4.0 bar and spacing of 0.20 m. Each dripper was connected to a multi‐outlet connector (model 802940) paired with four drip stakes (model 802850). All components were supplied by NaanDanJain Irrigation Company.

### 2.4 Water Replenishment

To determine the irrigation time required for water replenishment in each treatment, four pots were selected for each irrigation line. The water volumes required for the 100%, 50% and 25% treatments were calculated based on the difference between the reference weight at field capacity and the current weight of the pots on the day analysed, ensuring differentiation between treatments.

The irrigation time (Ti) was calculated using the mean of the four control pots according to the following equation:

$$\mathrm{Ti}=\frac{\mathrm{Vol}}{\mathrm{Ea}.q}$$

where Vol is the difference between the weight at field capacity and the current pot weight, in litres; Ea is the application efficiency of the system, expressed as a decimal; and *q* is the nominal flow rate of the drip stake (0.5 L·h^−1^).

Until 20 days after transplanting (DAT) the seedlings into the pots, all plants received a full water supply to meet total demand, with no differentiation in irrigation levels. After this period, different irrigation strategies were initiated to meet water replenishment levels of 100%, 50% and 25%. Irrigation was performed daily in the late afternoon. The volume of water to be replaced was determined individually for each pot based on daily weight measurements.

To replenish the water levels, four pots from each irrigation line were weighed on a daily basis. This procedure was performed for each irrigation level. Thus, the required water volume was defined as the difference between the current pot weight and the previous day's weight. For accuracy, the selected pots were weighed before and after each irrigation.

After assembling the irrigation system, uniformity tests were performed according to the methodology proposed by Keller and Karmeli (1974). The data collected in these tests were used to calculate the distribution uniformity coefficient (DUC) and Christiansen's uniformity coefficient (CUC). These tests were conducted at the beginning and end of the experiment to ensure the efficiency and consistency of the irrigation system. Additionally, humidity and temperature were continuously monitored throughout the experimental period in the greenhouses. This data was recorded using a data logger (RC‐51 model), as detailed in Figure S2.

For a better understanding of the experimental setup, seedlings were sown in trays on 4 October and transplanted into pots on 3 November, corresponding to 30 days after sowing. The experiment was conducted under controlled environmental conditions and had a total duration of 50 DAT. Morphological analyses, including plant height, number of leaves, number of leaflets, stem diameter, root growth, leaf dry mass and stem dry mass, were performed at 50 DAT. Physiological analyses, such as electrolyte leakage (EL), relative water content (RWC), membrane integrity (MI) and DNA content determination, were conducted at 35 DAT. Additionally, at 38 DAT, fully expanded leaves were collected for enzymatic activity assays, proline quantification and secondary metabolite analyses. Chlorophyll content was assessed at 40 DAT, before the onset of flowering.

### 2.5 Morphological Analyses

To assess vegetative growth, the following data were collected at 50 DAS: plant height and root growth (measured with a measuring tape), number of leaves and leaflets, stem diameter (measured with a digital calliper) and dry masses of leaves and stems.

For dry mass assessment, plant material was collected and dried in a forced‐air circulation oven at 40°C until a constant weight was achieved. The dried material was weighed using a precision analytical balance.

### 2.6 Physiological Analyses

Physiological analyses, such as EL, RWC and MI, were conducted at 35 DAT.

#### 2.6.1 EL

Four fragments, approximately 1 cm each, were excised from the middle‐third region of samples from each treatment and placed in Falcon tubes containing 20 mL of deionised water, with six replicates for each treatment. The tubes were agitated for 24 h at room temperature. After this period, the free electrical conductivity of the solution was measured.

The tubes were then placed in a water bath at 100°C for 1 h, and the total electrical conductivity of the solution was measured at the end of this step.

The EL rate was calculated using the formula proposed by Shi et al. (2006), as follows:

$$\mathrm{EL}\;\left(\%\right)=\frac{\mathrm{FEC}}{\mathrm{TEC}}\times 100$$

where FEC is the free electrical conductivity of the solution (after 24 h of agitation) and TEC is the total electrical conductivity of the solution (after heating at 100°C for 1 h).

#### 2.6.2 RWC

Four fragments of approximately 1 cm were excised from the middle third region of each sample. These fragments were weighed to quantify the fresh weight and placed in Falcon tubes containing deionised water, where they remained immersed for 24 h. After this period, the samples were reweighed to quantify the turgid weight and subsequently dried in a forced‐air circulation oven at 70°C for 48 h. Finally, the samples were weighed to determine their dry weights. RWC was calculated using the formula proposed by Barrs and Weatherley (1962), as follows:

$$\mathrm{RWC}\;\left(\%\right)=\frac{\left(\mathrm{FW}-\mathrm{DW}\right)}{\left(\mathrm{TW}-\mathrm{DW}\right)}\times 100$$

where FW is the fresh weight (g), TW is the turgid weight (g) and DW is the dry weight (g).

#### 2.6.3 MI

MI was estimated using a conductometer based on data obtained from the EL analysis, as described in Section 2.6.1. MI was calculated using the equation proposed by Azevedo et al. (2008), as follows:

$$\mathrm{MI}\;\left(\%\right)=\left(1-\left(\frac{\mathrm{FEC}}{\mathrm{TEC}}\right)\right)\times 100$$

where FEC is the free electrical conductivity (dS m^−1^) and TEC is the total electrical conductivity (dS m^−1^).

### 2.7 Biochemical Analyses

At 38 DAT, fully expanded leaves were collected for enzymatic activity analyses, proline quantification and secondary metabolite analyses. For enzymatic analyses, the leaves were immediately collected and stored in liquid nitrogen to preserve metabolic activity. For proline and secondary metabolite analyses, leaf samples were dried in a forced‐air circulation oven at 40°C until constant weight.

#### 2.7.1 Enzymatic Activity and Proline Content

To determine the activity of superoxide dismutase (SOD), catalase (CAT) and ascorbate peroxidase (APX), 0.2 g samples of fresh leaf tissue were ground in liquid N_2_ with polyvinylpolypyrrolidone (PVPP) following the methodology described by Biemelt et al. (1998). SOD activity was determined according to Giannopolitis and Ries (1977) and CAT activity was determined according to Havir and McHale (1987). For APX analysis, we adopted the protocol established by Nakano and Asada (1981).

For proline quantification, 0.2 g of dried leaves were homogenised in 80% methanol, kept under dark agitation for 24 h and subsequently centrifuged at 3000 rpm for 15 min. Supernatants were collected, and proline extraction was performed according to the method proposed by Bates et al. (1973).

### 2.8 Photosynthetic Pigments and DNA Content Analysis

The indices of chlorophyll a, chlorophyll b and total chlorophyll were quantified using an electronic chlorophyll metre (ClorofiLOG model CFL1030; Falker 2018).

Samples were collected 35 DAT for this analysis. DNA content was determined using flow cytometry. Samples containing 40–50 mg of leaf tissue from *L. lycopersicum* were finely chopped with a scalpel in a Petri dish containing 1 mL of Marie nucleus extraction buffer. After preparation, the samples were aspirated using a Pasteur pipette and filtered through a 50 μm mesh. The suspension was then stained with 25 μL of propidium iodide solution (1 mg mL^−1^). A total of 4000 nuclei from each sample were analysed for fluorescence emission (Marie and Brown 1993).

The analysis was performed using a BD FACSCalibur 4‐colour cytometer (Becton Dickinson). Histograms were generated and analysed using BD Cell Quest software (Figure S3). Each sample was analysed in triplicate. The reference standard was *Vicia faba* cv. Inovec (26.90 pg). Nuclear DNA content (pg) was estimated using the following equation:

$$\begin{array}{c} \mathrm{DNA}\;\text{Content}\;\left(\mathrm{pg}\right)\\ =\left(\text{G1 peak position of the sample}/\text{G1 peak position of}\;V.\;\text{faba}\right)\times 26.90. \end{array}$$

### 2.9 Secondary Metabolic Analyses

#### 2.9.1 Extraction and Quantification of Total Flavonoids and Total Phenolics

To determine the secondary metabolites, the extract was prepared as described for proline analysis in Section 2.7.1. The total flavonoid content (TFC) was determined using the method described by Ariza et al. (2016), in which 250 μL of hydro‐methanolic plant extract was mixed with 1.25 mL of deionised water and 75 μL of 5% sodium nitrate solution, followed by incubation for 6 min. Then, 500 μL of 10% aluminium chloride, 500 μL of 1 mol sodium hydroxide and 275 μL of water were added to the mixture. Absorbance was measured at 440 nm. A quercetin standard curve was prepared for the determination of flavonoids, and the flavonoid content was calculated based on this curve, with the results expressed as TFC (mg QE g^−1^).

To quantify the total phenolic compounds, 100 μL of plant extract was mixed with 500 μL of Folin–Ciocalteu solution and incubated in the dark for 3 min. Then, 400 μL of 7.5% sodium acetate was added, and the samples were incubated in the dark for 2 h. The absorbance was measured at 760 nm. The total phenolic content was determined using gallic acid as a reference standard. A standard curve was prepared using different concentrations, and the results were expressed as milligrams of gallic acid equivalents per gram of dry matter (mg GAE/g^−1^), following the methodologies established by Chang et al. (2002) and Memari‐Tabrizi et al. (2021).

### 2.10 Statistical Analysis

The data obtained were subjected to analysis of variance (ANOVA) using the *F*‐test at 1% and 5% levels of significance. Treatment means were compared using Tukey's test (*p* < 0.05). The analyses were conducted using R software, version 4.1.2 (R Core Team 2009), specifically with the ExpDes.pt.library, version 1.2.2 (Ferreira et al. 2021).

## 3 Results

### 3.1 Morphological Analyses

**FIGURE 1:**
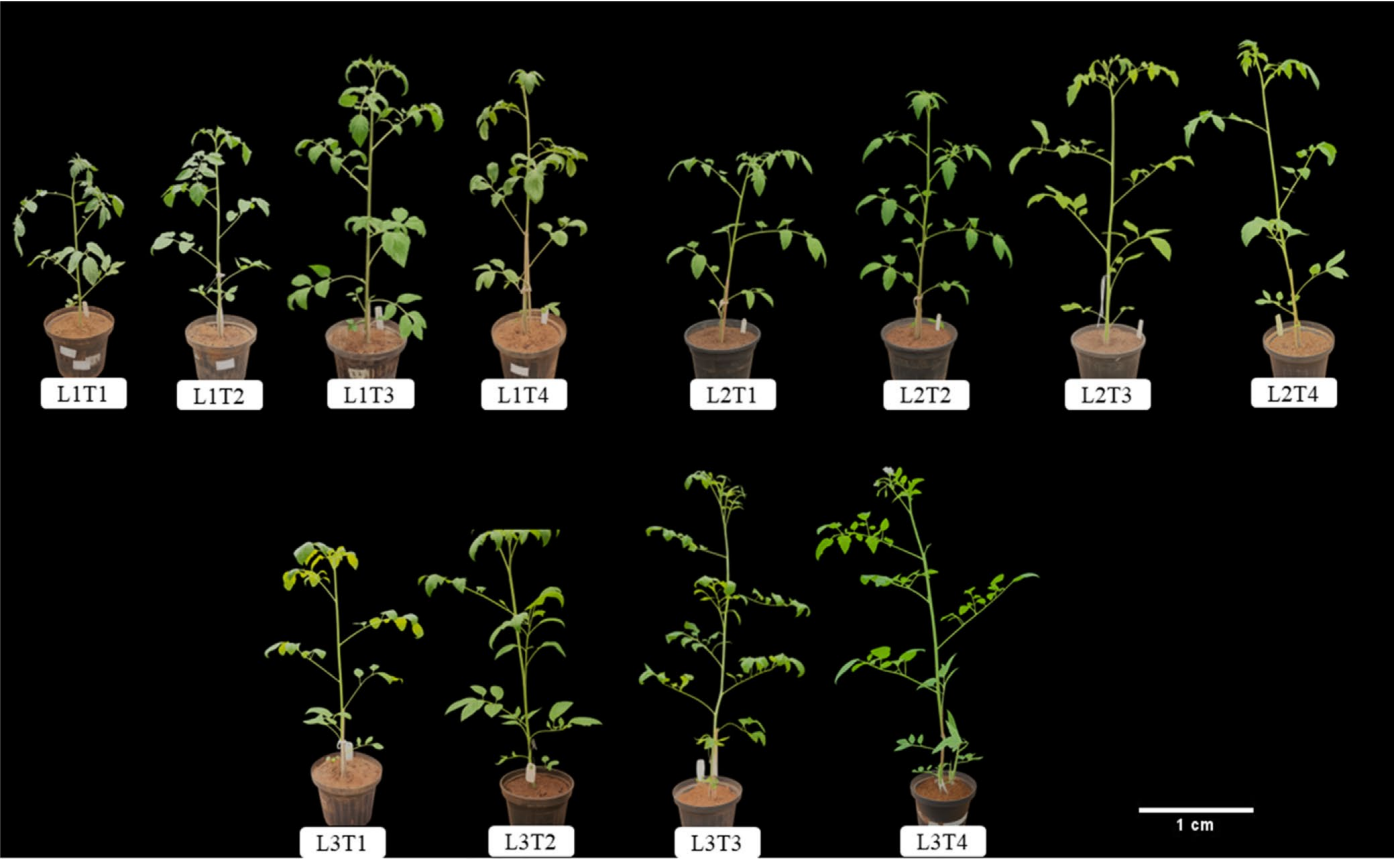
Evaluation of *Solanum lycopersicum* under different irrigation levels (25%, 50% and 100%) and treatments with *Azospirillum brasilense* and *Bacillus amyloliquefaciens* , as well as co‐inoculation of both bacteria. L1, 25% replenishment line; L2, 50% replenishment line; L3, 100% replenishment line; T1, control treatment without bacteria; T2, treatment with *A. brasilense* ; T3, treatment with *B. amyloliquefaciens* ; and T4, treatment with a combined application of *A. brasilense* and *B. amyloliquefaciens* .

Growth parameters were analysed to evaluate the responses of the plants to different irrigation systems and their interactions with PGPB (Figure 1).

**FIGURE 2:**
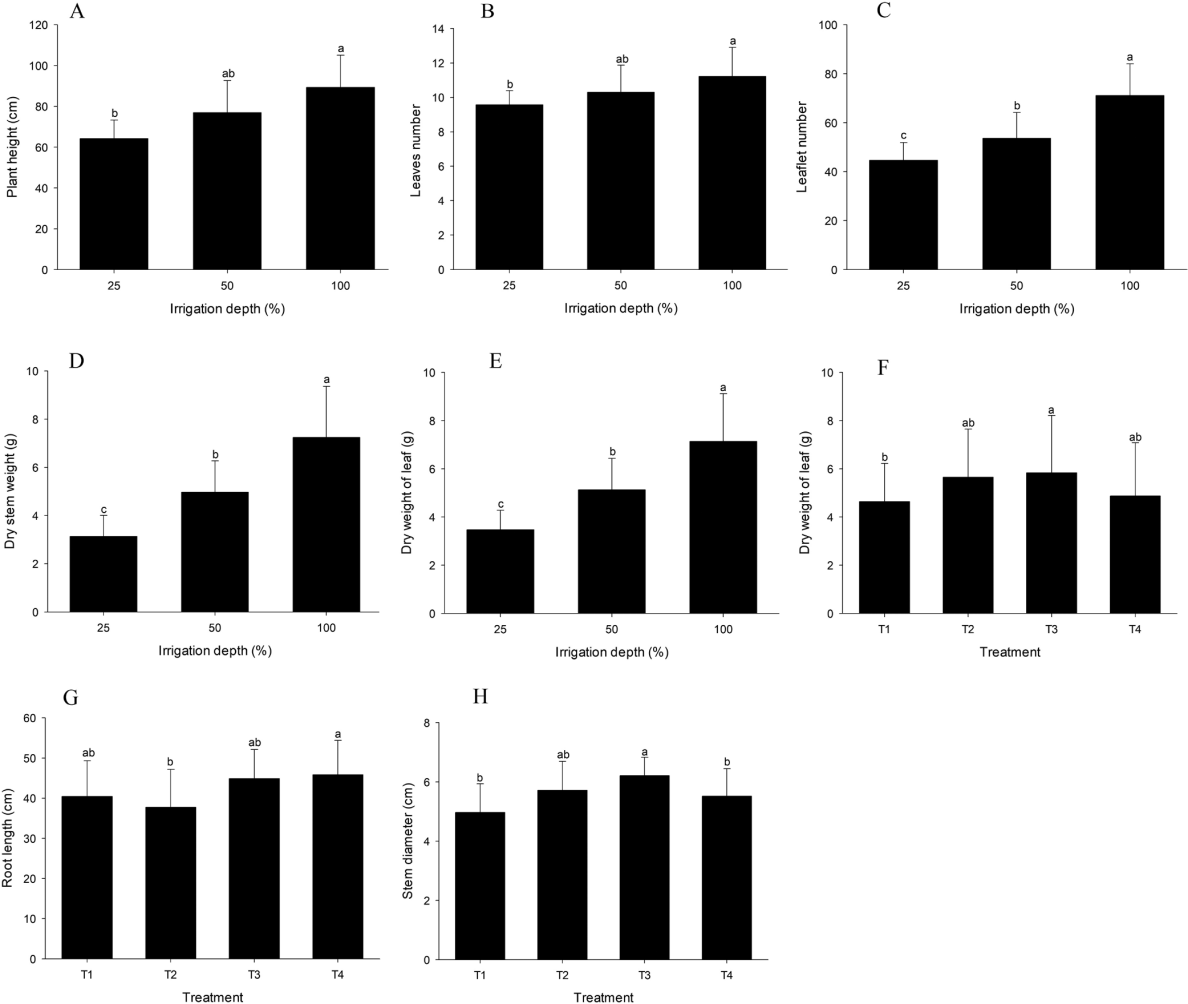
Morphological parameters of *Solanum lycopersicum* under different irrigation levels (25%, 50% and 100%) and treatments with *Azospirillum brasilense* and *Bacillus amyloliquefaciens* , as well as co‐inoculation with both bacteria. (A) Plant height (cm), (B) number of leaves, (C) number of leaflets, (D) stem dry mass, (E) leaf dry mass for the irrigation treatment, (F) leaf dry mass for the bacterial treatment, (G) root length and (H) stem diameter. T1, control treatment without bacteria; T2, treatment with *A. brasilense* ; T3, treatment with *B. amyloliquefaciens* ; and T4, treatment with the combined application of *A. brasilense* and *B. amyloliquefaciens* . Bars followed by the same letter do not differ significantly at the 5% significance level according to Tukey's test.

The plant height, number of leaves and leaflets and stem dry mass variables differed statistically only because of different irrigation levels (Figure 2A–C) and were not influenced by treatment with *A. brasilense* and *B. amyloliquefaciens* , nor by the interaction between bacteria and irrigation levels (Table S1). In this context, plant height and number of leaves followed the same pattern, with the highest values observed under the 100% irrigation level differing significantly only from the 25% irrigation level. However, the number of leaflets showed statistically significant differences across the three irrigation levels (Figure 2C).

No significant statistical interaction was observed between irrigation levels and bacterial treatments for the variable leaf dry mass (Table S1). However, differences were observed in the main effects of irrigation and bacteria independently (Figure 2D). When assessed for irrigation levels, lower dry mass accumulation was observed under water restriction (25% irrigation level), whereas the highest accumulation occurred with the highest water availability (100% irrigation level) (Figure 2E). Regarding microorganism inoculation, only the inoculation with *B. amyloliquefaciens* (T3) showed a statistically significant difference compared to the control (T1), exhibiting higher leaf dry mass content (Figure 2F).

Similar to other morphological traits, stem diameter and root growth variables (Figure 2G,H) did not show significant interaction effects between the irrigation levels and bacterial treatments (Table S2). However, significant differences were observed when the bacterial treatments were considered independently. Stem diameter was larger in the inoculation with *B. amyloliquefaciens* (T3) than in the control (T1). In contrast, root length was greatest under the co‐inoculation treatment with *B. amyloliquefaciens* and *A. brasilense* (T4) compared to treatment with *A. brasilense* alone (T2).

### 3.2 Physiological Analyses

**FIGURE 3:**
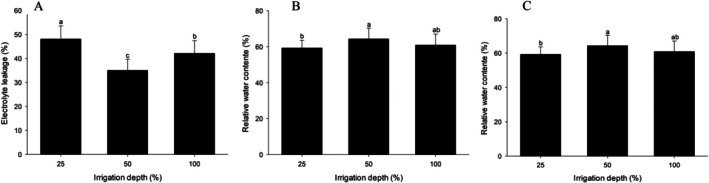
Physiological parameters of *Solanum lycopersicum* under different irrigation levels (25%, 50% and 100%) and treatments with *Azospirillum brasilense* and *Bacillus amyloliquefaciens* , as well as co‐inoculation of both bacteria. (A) Electrolyte leakage, (B) relative water content and (C) membrane integrity. Bars followed by the same letter do not differ significantly at the 5% significance level according to Tukey's test.

Physiological analyses, characterised by El, RWC and MI, revealed differences only because of irrigation (Table S3). EL was higher under water‐restricted conditions than under the other treatments (Figure 3A). The variable RWC showed a significant difference at the 50% irrigation level when compared to the 25% water restriction level, where plants receiving 50% water replenishment exhibited higher means than those subjected to 25% water restriction. Plants subjected to full irrigation (100%) did not differ significantly from those subjected to 25% and 50% irrigation (Figure 3B). The highest MI was observed in plants treated with a 50% irrigation level, whereas the 25% replenishment level exhibited the lowest MI (Figure 3C).

### 3.3 Biochemical Analyses

The enzymatic analysis assessed the activity of SOD, CAT and APX. Significant differences in enzymatic activity were observed when considering the interaction between irrigation and bacterial treatments (Table 2). For the SOD enzyme, among the irrigation levels within each bacterial treatment, only *B. amyloliquefaciens* inoculation (T3) presented a significant response to the water regime, with a decrease in SOD production observed at the 50% irrigation level when compared to the 25% irrigation level. Among the inoculation treatments, only the co‐inoculation of *A. brasilense* and *B. amyloliquefaciens* (T4) showed higher values than the control (T1) at the 25% irrigation level. At 50% water replenishment, only *B. amyloliquefaciens* inoculation (T3) showed significantly lower values than the control (T1). For full irrigation (100%), no significant differences were observed between the treatments (*p* > 0.05) (Table 2).

**TABLE 2:** Parameters of *Solanum lycopersicum* under different irrigation levels (25%, 50% and 100%) and treatments with *Azospirillum brasilense* and *Bacillus amyloliquefaciens* , as well as co‐inoculation with both bacteria. T1, control treatment without bacteria; T2, treatment with *A. brasilense* ; T3, treatment with *B. amyloliquefaciens* ; T4, treatment with a combined application of *A. brasilense* and *B. amyloliquefaciens* .

Enzymes	Treatments	Irrigation depth		
		25%	50%	100%
SOD (U min^−1^ mg^−1^ of protein)	T1	215.21Ab	267.79Aa	208.75Aa
	T2	185.71Ab	202.05Aab	180.46Aa
	T3	233.27Aab	126.81Bb	158.57ABa
	T4	314.27Aa	206.04Bab	209.36Ba
CAT (μM H_2_O_2_ min^−1^ mg^−1^ of FW)	T1	0.802Aab	0.542Bb	0.480Ba
	T2	0.942Aa	0.417Bb	0.544Ba
	T3	0.554Ab	0.436Ab	0.566Aa
	T4	0.547Bb	0.831Aa	0.613Ba
APX (μM H_2_O_2_ min^−1^ mg^−1^ of FW)	T1	8.527Ab	8.507Aa	6.600Aa
	T2	11.900Aa	5.751Bab	5.569Ba
	T3	6.792Abc	5.036Ab	4.659Aa
	T4	3.823Ac	5.954Aab	3.671Aa

*Note:* Means followed by the same letters in the columns do not differ from each other by Tukey's test at a 5% significance level. Lowercase letters for bacterial inoculation and uppercase letters for irrigation level.

For the CAT enzyme, *A. brasilense* inoculation (T2) did not differ from the control (T1); however, it showed a higher value compared to the co‐inoculation of *A. brasilense* and *B. amyloliquefaciens* (T4) and *B. amyloliquefaciens* inoculation (T3) at the 25% irrigation level.

The co‐inoculation of *A. brasilense* and *B. amyloliquefaciens* (T4) resulted in the highest CAT production at 50% replenishment, with lower production at both 25% and 100% irrigation. The *B. amyloliquefaciens* inoculation (T3) showed no significant difference between the irrigation levels. With 25% water replenishment, all treatments showed no significant difference compared to the control (T1). With 50% water replenishment, the co‐inoculation of *A. brasilense* and *B. amyloliquefaciens* (T4) resulted in the highest CAT production. No statistical differences were observed between the treatments at the 100% irrigation level (Table 2).

When quantifying APX, it was observed that irrigation strategies had a limited influence on the treatments between the irrigation levels. At the 25% irrigation level, *A. brasilense* inoculation (T2) exhibited the highest APX activity (*p* < 0.05). However, at the 50% water replenishment level, *A. brasilense* (T2) and *B. amyloliquefaciens* (T3) inoculation were statistically similar to the control (T1), whereas the co‐inoculation (T4) was significantly lower than the control. At the 100% irrigation level, no significant differences were observed between the treatments (*p* > 0.05) (Table 2).

**FIGURE 4:**
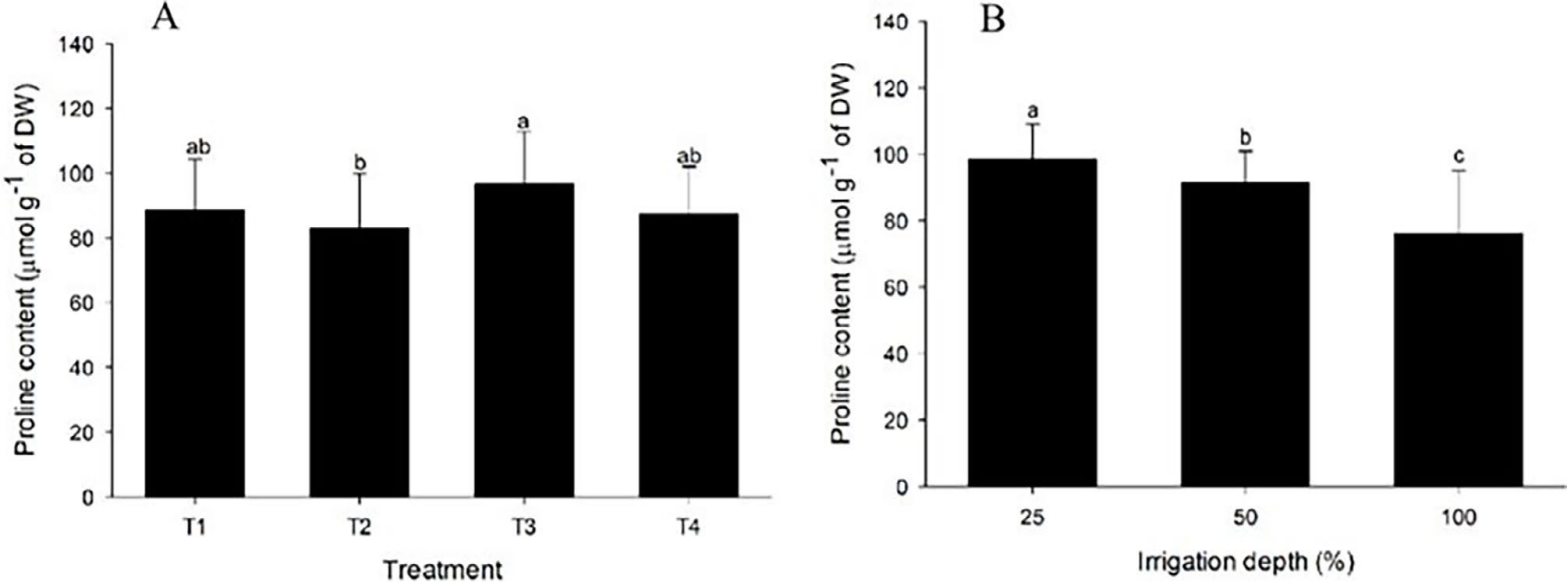
Proline parameters of *Solanum lycopersicum* under different irrigation levels (25%, 50% and 100%) and treatments with *Azospirillum brasilense* and *Bacillus amyloliquefaciens* , as well as co‐inoculation with both bacteria. Proline content under different irrigation levels and proline content under different bacterial inoculations. T1, control treatment without bacteria; T2, treatment with *A. brasilense* ; T3, treatment with *B. amyloliquefaciens* ; T4, treatment with a combined application of *A. brasilense* and *B. amyloliquefaciens* . Means followed by the same letters in the columns do not differ from each other by Tukey's test at a 5% significance level.

Proline quantification showed variation only for the main effects of irrigation and bacterial treatments when evaluated separately (Table S4). Regarding irrigation levels, there was a higher accumulation of proline under water restriction (25% replenishment), and this accumulation decreased as the irrigation level increased (Figure 4B). No statistically significant differences were observed in the microorganisms compared to the control (T1) for any bacterial inoculation treatment. However, *A. brasilense* inoculation (T2) resulted in lower proline levels than *B. amyloliquefaciens* inoculation (T3) (Figure 4A).

### 3.4 Photosynthetic Pigments and DNA Content Analysis

**FIGURE 5:**
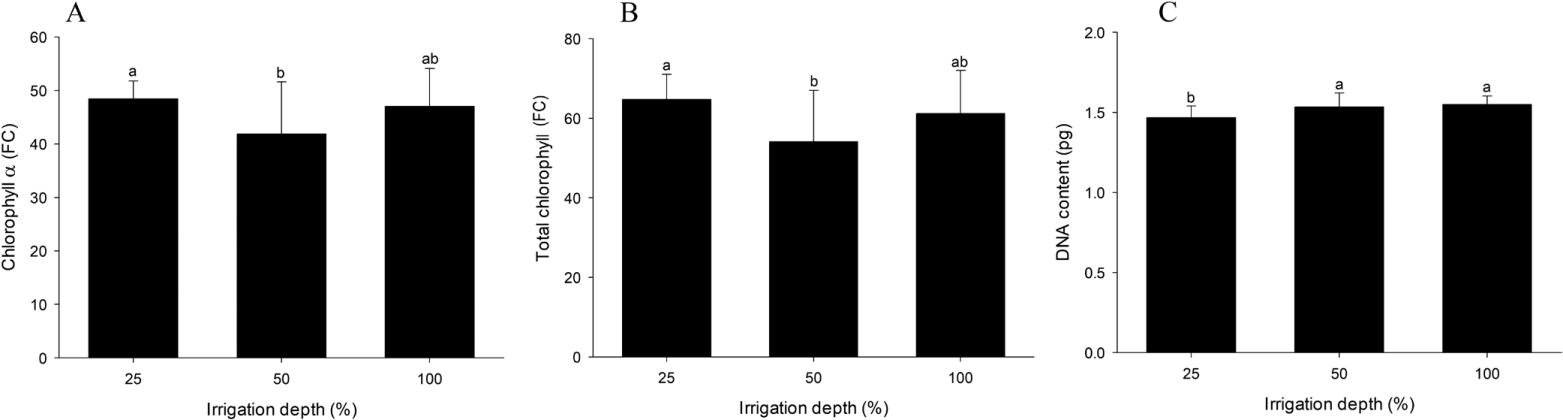
Photosynthetic pigment parameters and DNA content of *Solanum lycopersicum* under different irrigation levels (25%, 50% and 100%). (A) Chlorophyll a index, (B) total chlorophyll index and (C) DNA content. Bars followed by the same letters do not differ significantly at the 5% significance level according to Tukey's test.

For chlorophyll α and total chlorophyll, significant differences were observed only between the different irrigation levels (Table S5). Higher levels of chlorophyll α and total chlorophyll were recorded at the 25% water replenishment level compared to the 50% irrigation level (Figure 5A,B).

The variable DNA content differed only between irrigation levels (Table S5). The 25% water replenishment level presented the lowest DNA content, differing significantly from the 50% and 100% levels, which did not vary among themselves (Figure 5C).

### 3.5 Secondary Metabolism Analyses

Flavonoid quantification was influenced by the interaction between irrigation and bacterial treatments (Table S6). Among the irrigation levels, differences were observed only for treatments with *A. brasilense* inoculation (T2) and the co‐inoculation of *A. brasilense* and *B. amyloliquefaciens* (T4), which displayed specific effects per treatment. The treatment with *A. brasilense* inoculation (T2) showed higher flavonoid content at the 25% water replenishment level and lower means at the 50% level. For the co‐inoculation of *A. brasilense* and *B. amyloliquefaciens* (T4), higher values were recorded at the 100% water replenishment level than at the other levels, whereas lower values were observed at the 50% irrigation level. At the 25% water replenishment level, no differences were observed between any of the treatments and the control (T1). At the 50% water replenishment level, only the co‐inoculation of *A. brasilense* and *B. amyloliquefaciens* (T4) showed significantly lower flavonoid content than the control (T1). Under full irrigation (100% water replenishment), only the co‐inoculation of *A. brasilense* and *B. amyloliquefaciens* (T4) showed higher flavonoid content compared to the control (T1) (Table 3).

**TABLE 3:** Secondary metabolite parameters of *Solanum lycopersicum* under different irrigation levels (25%, 50% and 100%) and treatments with *Azospirillum brasilense* and *Bacillus amyloliquefaciens* . T1, control treatment without bacteria; T2, treatment with *A. brasilense* ; T3, treatment with *B. amyloliquefaciens* ; T4, treatment with a combined application of *A. brasilense* and *B. amyloliquefaciens* .

Metabolites	Treatments	Irrigation depth		
		25%	50%	100%
Flavonoid (TFC mg QE g^−1^)	T1	19.763Aab	17.752Aab	16.588Ab
	T2	22.311Aa	14.163Cbc	18.484Bab
	T3	17.390Ab	19.018Aa	17.676Aab
	T4	17.223Bb	13.438Cc	20.730Aa
Total phenolics (mg GAE g^−1^ of DW)	T1	4.273Aab	3.921Aa	3.445Ab
	T2	4.919Aa	3.450Ba	3.549Bb
	T3	3.318Ab	3.855Aa	3.398Ab
	T4	3.932Bab	3.962Ba	5.824Aa

*Note:* Means followed by the same letters in the columns do not differ from each other by Tukey's test at a 5% significance level. Lowercase letters for bacterial inoculation and uppercase letters for irrigation level.

The interaction between irrigation and bacterial treatments was significant for the quantification of total phenolics (Table S6). The effect of irrigation was mainly observed in the co‐inoculation treatment with *A. brasilense* and *B. amyloliquefaciens* (T4), in which the highest phenolic content was recorded under 100% irrigation. At the 25% irrigation level, the treatment with *A. brasilense* (T2) showed distinct values compared with the others, while the remaining treatments did not differ from the control (T1). The co‐inoculation with *A. brasilense* and *B. amyloliquefaciens* (T4) resulted in the highest phenolic production at 100% water replenishment (Table 3).

## 4 Discussion

Tomato crops are highly susceptible to water scarcity at various developmental stages, from germination to fruit formation, which can induce molecular, morphological, physiological and cellular changes (Bacallao and Fundora 2014). This study evaluated the response of *S. lycopersicum* to severe water stress (25% water replenishment) and the intervention of PGPB, highlighting important plant adaptations and the potential of bioinoculants.

Morphological analyses revealed that severe water stress significantly compromised tomato growth, negatively affecting plant height, leaf and leaflet numbers and dry mass of stems and leaves (Figure 2). This reduction in growth is a well‐documented physiological response, as plant growth fundamentally depends on cellular expansion, which requires water uptake and maintenance of turgor pressure. Under water‐deficient conditions, water uptake is insufficient, limiting cell elongation and, consequently, overall plant development, as noted by Hsiao and Acevedo (1975), Matos (2020) and Cruz et al. (2021). Similar results were reported by Tüzel et al. (2025), who found that water stress significantly reduced tomato plant growth, with a reduction of up to 27% in plant height and also affected the dry weight of vegetative structures compared to full irrigation.

Morphological analyses revealed that, under severe water deficit, inoculation with *B. amyloliquefaciens* and more notably co‐inoculation with *A. brasilense* , resulted in significant increases in dry mass, stem diameter and root development compared to non‐inoculated plants (Figure 2). This mitigating effect can be mechanistically attributed to the known ability of *A. brasilense* to promote root growth through the production of phytohormones such as auxins and gibberellins (Etesami 2025). Auxins, in particular, are crucial for root elongation and branching, expanding the soil exploration surface and, consequently, the capacity for water and nutrient absorption. Additionally, *B. amyloliquefaciens* is recognised for its ability to solubilise nutrients and synthesise cytokinins, which regulate cell division and expansion, contributing to increased dry mass and stem diameter (Beltrán‐Acosta et al. 2023). The synergy observed in co‐inoculation (T4) suggests that the different growth‐promoting strategies of both bacteria (such as the improvement of root architecture by *A. brasilense* and the increased availability of nutrients and production of other growth factors by *B. amyloliquefaciens* ) confer an additive benefit, optimising the plant's ability to sustain growth even under severe water stress conditions. Other microorganisms, such as *Bacillus subtilis* (Pei et al. 2021), *Pseudomonas* sp. and *Bacillus thuringiensis* (Rojas‐Solís et al. 2016), have also been associated with improvements in tomato crop growth parameters.

Physiological analyses, including assessments of electrolyte leakage (EL), RWC and MI, are crucial for evaluating plant cellular health. The results indicated that severe water stress led to a significant increase in EL and a decrease in MI and RWC (Figure 3). Elevated EL is a biomarker of plasma membrane damage, resulting from stability loss and increased permeability, commonly associated with reactive oxygen species (ROS) accumulation under stress conditions (Demidchik et al. 2014; Khoshbakht and Asgharei 2015; Javadipour et al. 2022). Maintaining MI is essential for cellular homeostasis and selective ion transport (Wijewardana et al. 2016; Altinci and Cangil 2019). The findings of this study regarding the reduced RWC and MI under severe stress align with previous observations in tomatoes by Patanè et al. (2022) and Ors et al. (2021). Similar responses have been documented in lettuce (De Souza Freitas et al. 2019) and rice (Ali et al. 2023), where water stress increased EL Table and reduced RWC.

Biochemical analyses were used to evaluate the activities of the antioxidant enzymes SOD, CAT and APX, as well as proline quantification (Figure 4). Water stress induced an increase in the production of these enzymes and proline accumulation, demonstrating a plant defence response to oxidative stress. ROS formation is a consequence of water stress, and SOD is one of the first enzymes in the defence mechanism that converts superoxide radicals into hydrogen peroxide (Dat et al. 2000; Hussain et al. 2016; Novello et al. 2020). CAT and APX play critical roles in detoxifying hydrogen peroxide (Caverzan et al. 2016; Ghanbarzadeh et al. 2019; Lahbouki et al. 2022; Barbosa et al. 2023). Proline, which acts as both an osmoprotectant and an oxidative damage protector, accumulated more under severe water restriction, indicating the level of stress and the plant's effort to maintain homeostasis (Hatzing et al. 2014; Harsh et al. 2016; Weltmeier et al. 2006; Sharma and Dubey 2005).

The effects of bacterial inoculation varied in terms of enzymatic activity and proline levels. For instance, co‐inoculation and inoculation with *B. amyloliquefaciens* led to higher SOD levels under certain stress conditions (Figure 4). In *Solanum melongena* L., water‐stressed plants inoculated with *Azotobacter chroococcum* and *Azotobacter vinelandii* showed significant increases in SOD and CAT activity compared to the controls (Kiran et al. 2022). Similar results were reported for *S. lycopersicum* inoculated with *B. subtilis* under water stress (Gowtham et al. 2020). However, in *Oryza sativa* L. under saline stress, inoculation with *B. amyloliquefaciens* significantly reduced proline content compared to non‐inoculated plants (Bisht et al. 2025), highlighting the complexity and specificity of plant–microbe interactions.

The results revealed higher chlorophyll a and total chlorophyll levels in plants subjected to 25% water replenishment (Figure 5). This increase may represent an adaptive response in which the plant compensates for oxidative stress damage by maximising light capture to sustain photosynthesis (Dbira et al. 2018; De Lima et al. 2020). However, the plant's response to chlorophyll content may depend on the type and intensity of the stress. For example, tomato plants exposed to saline stress showed a 40% reduction in total chlorophyll content (Shahzadi et al. 2025), whereas high‐temperature conditions increased chlorophyll a production (Soengas et al. 2018).

The reduced DNA content in tomato plants irrigated under the lowest water replenishment level (25%) (Figure 5) is consistent with previous reports highlighting the impact of water stress on genomic stability and cellular processes in plants. According to Manivannan et al. (2007), severe water deficit in *Helianthus annuus* L. is often accompanied by increased ROS production, which can result in DNA damage, including single‐ and double‐strand breaks, directly impairing the plant's ability to sustain replication and cell division. Additionally, Shinozaki and Yamaguchi‐Shinozaki (2007) noted that water deficit activates cellular stress response pathways that modulate the cell cycle, often halting the G1‐to‐S phase transition, which may explain the reduced DNA content under conditions of severe stress. These alterations are strongly correlated with excessive ROS production, which not only damages DNA molecules but also disrupts the cell cycle and repair mechanisms, severely compromising genomic integrity (Abdelhameed et al. 2024; Whitney et al. 2019).

Secondary metabolite analyses showed that flavonoid quantification was influenced by the interaction between irrigation level and bacterial treatments, with the highest levels observed in the *A. brasilense* inoculation (T2) under 25% water replenishment. For total phenolics, the co‐inoculation of *A. brasilense* and *B. amyloliquefaciens* (T4) resulted in the highest phenolic content under 100% irrigation. Both secondary metabolites are part of a non‐enzymatic antioxidant defence system that is crucial for protecting plants against abiotic stresses (Tohidi et al. 2017; Jan et al. 2022). Flavonoids accumulate under water stress and function in ROS scavenging via redox reactions (Treutter 2006; Nakabayashi et al. 2014; Foyer and Noctor 2005). This increase in secondary metabolite production serves as a critical water stress protection mechanism, neutralising free radicals and stimulating the activity of antioxidant enzymes (Valifard et al. 2014; Qari and Tarbiyyah 2021). Increased secondary metabolites under stress have also been reported in tomatoes and other species, such as eggplants, under water stress (Xu et al. 2024; Kiran et al. 2022).

## 5 Conclusion

This study highlights the significant impact of severe water stress (25% water replenishment) on tomato plants, compromising their growth, cellular integrity and genomic stability. However, the use of PGPB, such as *B. amyloliquefaciens* and *A. brasilense* , has proven to be a promising strategy for mitigating these negative effects, promoting greater dry mass accumulation, enhancing antioxidant enzyme activity and regulating secondary metabolite production. These findings underscore the importance of integrated management practices, combining biotechnological strategies and water supply adjustments tailored to crop requirements, to improve plant tolerance to water deficits and contribute to agricultural sustainability in the context of climate change in the future.

## Author Contributions

**Camila Garcia de Freitas:** investigation, methodology, data curation, formal analysis, writing – review and editing, writing – original draft, validation. **Mádilo Lages Vieira Passos:** methodology, data curation, formal analysis, software. **Mariana de Vasconcelos Dias:** methodology, validation. **Leandro Israel da Silva:** methodology, validation. **Lilian Ferreira de Sousa:** writing – review and editing. **Filipe Almendagna Rodrigues:** methodology, validation. **Caroline Dambroz:** supervision, project administration, writing – review and editing. **Vytória Piscitelli Cavalcanti:** visualization, methodology, data curation, writing – review and editing. **Joyce Dória:** conceptualization, writing – review and editing, resources, funding acquisition.

## Conflicts of Interest

The authors declare no conflicts of interest.

## Supporting information


**Table S1:** Summary of variance analysis with degrees of freedom (DF) and mean squares for the variables: Plant height (PH), number of leaves (NL), number of leaflets (NFO), stem dry mass (SDM) and leaf dry mass (LDM) of *Solanum lycopersicum* under different irrigation.
**Table S2:** Summary of the analysis of variance with degrees of freedom (DF) and mean squares for the variables stem diameter (SD) and root growth (RG) of *Solanum lycopersicum* under different irrigation levels (25%, 50% and 100%) and treatments with *Azospirillum brasilense* and *Bacillus subtilis* , as well as co‐inoculation with both bacteria.
**Table S3:** Summary of the analysis of variance with degrees of freedom (DF) and mean squares for the variables Relative water content (RWC), electrolyte leakage (EL) and membrane integrity (MI) of *Solanum lycopersicum* under different irrigation levels (25%, 50% and 100%) and treatments with *Azospirillum brasilense* and *Bacillus subtilis* , as well as co‐inoculation with both bacteria.
**Table S4:** Summary of variance analysis with degrees of freedom (DF) and mean squares for the variables: Superoxide dismutase (SOD), catalase (CAT), ascorbate peroxidase (APX) and proline of *Solanum lycopersicum* under different irrigation levels (25%, 50% and 100%) and treatments with *Azospirillum brasilense* and *Bacillus amyloliquefaciens* , as well as co‐inoculation with both bacteria.
**Table S5:** Summary of the analysis of variance with degrees of freedom (DF) and mean squares for the variables chlorophyll A (CLA), chlorophyll B (CLB), total chlorophyll (CLT) and DNA content (CDNA) of *Solanum lycopersicum* under different irrigation levels (25%, 50% and 100%) and treatments with *Azospirillum brasilense* and *Bacillus amyloliquefaciens* , as well as co‐inoculation with both bacteria.
**Table S6:** Summary of the analysis of variance with degrees of freedom (DF) and mean squares for the variables: Flavonoids and total phenolic compounds (phenols) of *Solanum lycopersicum* under different irrigation levels (25%, 50% and 100%) and treatments with *Azospirillum brasilense* and *Bacillus amyloliquefaciens* , as well as co‐inoculation with both bacteria.

**Figure S1:** Experimental design of potted tomato plants, distributed by blocks (5) and treatments (irrigation levels × bacteria). L1: line with 25% blade replacement; L2: line with 50% blade replacement; L3: line with 100% blade replacement; T1: control treatment without bacteria; T2: treatment with *Azospirillum brasiliense*; T3: treatment with *Bacillus amyloliquefaciens* ; and T4: treatment with a combination of *Azospirillum brasiliense* and *Bacillus amyloliquefaciens* ; P1: Plant 1 and P2: Plant 2.
**Figure S2:** Temporal variation of temperature (°C) and relative air humidity (%) monitored in the experimental environment during the study period.
**Figure S3:** Fluorescence histograms by flow cytometry after staining with propidium iodide. (A) Histogram for treatment with 25% slide replacement; (B) histogram for treatment with 50% slide replacement and (C) histogram for treatment with 100% slide replacement. The *x*‐axis represents the fluorescence intensity (reflecting the amount of stained DNA in each nucleus), while the *y*‐axis shows the cell count (frequency of occurrence).

## Data Availability

Data sharing is not applicable to this article as no datasets were generated or analysed during the current study.
