# Supplementary material for: Inoculation With Azospirillum brasilense and Bacillus amyloliquefaciens Enhances Tomato Resilience to Severe Water Deficit: A Comprehensive Morpho‐Physiological and Biochemical Analysis
Source: Environ Microbiol Rep. 2026 Apr 28;18(3):e70316. doi: 10.1111/1758-2229.70316 (PMC13124666; doi:10.1111/1758-2229.70316)
Supplement: Supplementary file 1 — Table S1: Summary of variance analysis with degrees of freedom (DF) and mean squares for the variables: Plant height (PH), number of leaves (NL), number of leaflets (NFO), stem dry mass (SDM) and leaf dry mass (LDM) of Solanum lycopersicum under different irrigation. Table S2: Summary of the analysis of variance with degrees of freedom (DF) and mean squares for the variables stem diameter (SD) and root growth (RG) of Solanum lycopersicum under different irrigation levels (25%, 50% and 100%) and treatments with Azospirillum brasilense and Bacillus subtilis , as well as co‐inoculation with both bacteria. Table S3: Summary of the analysis of variance with degrees of freedom (DF) and mean squares for the variables Relative water content (RWC), electrolyte leakage (EL) and membrane integrity (MI) of Solanum lycopersicum under different irrigation levels (25%, 50% and 100%) and treatments with Azospirillum brasilense and Bacillus subtilis , as well as co‐inoculation with both bacteria. Table S4: Summary of variance analysis with degrees of freedom (DF) and mean squares for the variables: Superoxide dismutase (SOD), catalase (CAT), ascorbate peroxidase (APX) and proline of Solanum lycopersicum under different irrigation levels (25%, 50% and 100%) and treatments with Azospirillum brasilense and Bacillus amyloliquefaciens , as well as co‐inoculation with both bacteria. Table S5: Summary of the analysis of variance with degrees of freedom (DF) and mean squares for the variables chlorophyll A (CLA), chlorophyll B (CLB), total chlorophyll (CLT) and DNA content (CDNA) of Solanum lycopersicum under different irrigation levels (25%, 50% and 100%) and treatments with Azospirillum brasilense and Bacillus amyloliquefaciens , as well as co‐inoculation with both bacteria. Table S6: Summary of the analysis of variance with degrees of freedom (DF) and mean squares for the variables: Flavonoids and total phenolic compounds (phenols) of Solanum lycopersicum under different irrigation level [file EMI4-18-e70316-s002.docx]

**SUPPLEMENTARY MATERIAL – TABLES**

**Table S1** – Summary of variance analysis with degrees of freedom (DF) and mean squares for the variables: Plant Height (PH), Number of Leaves (NL), Number of Leaflets (NFO), Stem Dry Mass (SDM), and Leaf Dry Mass (LDM) of *Solanum lycopersicum* under different irrigation

levels (25%, 50%, and 100%) and treatments with *Azospirillum brasilense* and *Bacillus amyloliquefaciens*, as well as co-inoculation with both bacteria.

| Source of variation | DF | PH | NL | NFO | SDM | LDM |
| --- | --- | --- | --- | --- | --- | --- |
| Water regime (WR) | 2 | 13.8146 ** | 9.0441** | 49.778** | 84,932** | 67,306** |
| Bacteria (B) | 3 | 0.5281 ^NS^ | 1.9407 ^NS^ | 2.026 ^NS^ | 1,766^NS^ | 5,052* |
| WR x B | 6 | 0.6138 ^NS^ | 0.5973 ^NS^ | 0.809 ^NS^ | 2,234^NS^ | 15,808^NS^ |

*Significant at 5%; **Significant at 1%; and NS Not significant.

**Table S2** – Summary of the analysis of variance with degrees of freedom (DF) and mean squares for the variables Stem Diameter (SD) and Root Growth (RG) of *Solanum lycopersicum* under different irrigation levels (25%, 50%, and 100%) and treatments with *Azospirillum brasilense* and *Bacillus subtilis,* as well as co-inoculation with both bacteria.

| Source of variation | DF | SD | RG |
| --- | --- | --- | --- |
| Water regime (WR) | 2 | 1.4492 ^NS^ | 0.3773 ^NS^ |
| Bacteria (B) | 3 | 5.9566** | 4.8729** |
| WR x B | 6 | 1.6842 ^NS^ | 0.9893 ^NS^ |

*Significant at 5%; **Significant at 1%; and NS Not significant.

**Table S3** – Summary of the analysis of variance with degrees of freedom (DF) and mean squares for the variables Relative water content (RWC), Electrolyte leakage (EL), and Membrane integrity (MI) of *Solanum lycopersicum* under different irrigation levels (25%, 50%, and 100%) and treatments with *Azospirillum brasilense* and *Bacillus subtilis*, as well as co-inoculation with both bacteria.

| Source of variation | DF | RWC% | EL% | MI% |
| --- | --- | --- | --- | --- |
| Water regime (WR) | 2 | 5.2872* | 85.812*** | 33.552*** |
| Bacteria (B) | 3 | 0.9419 ^NS^ | 1.030 ^NS^ | 1.759 ^NS^ |
| WR x B | 6 | 0.6729 ^NS^ | 1.479 ^NS^ | 1.603 ^NS^ |

*Significant at 5%; **Significant at 1%; and NS Not significant.

**Table S4** – Summary of variance analysis with degrees of freedom (DF) and mean squares for the variables: Superoxide Dismutase (SOD), Catalase (CAT), Ascorbate Peroxidase (APX), and Proline of *Solanum lycopersicum* under different irrigation levels (25%, 50%, and 100%) and treatments with *Azospirillum brasilense* and *Bacillus amyloliquefaciens*, as well as co-inoculation with both bacteria.

| Source of variation | DF | SOD | CAT | APX | Proline |
| --- | --- | --- | --- | --- | --- |
| Water regime (WR) | 2 | 12484.24^NS^ | 0.165** | 34.854** | 2590.976** |
| Bacteria (B) | 3 | 16627.24** | 0.0602^NS^ | 42.371** | 486.798** |
| WR x B | 6 | 9091.16* | 0.166*** | 16.947** | 290.632NS |

*Significant at 5%; **Significant at 1%; and NS Not significant.

**Table S5** – Summary of the analysis of variance with degrees of freedom (DF) and mean squares for the variables Chlorophyll A (CLA), Chlorophyll B (CLB), Total Chlorophyll (CLT), and DNA Content (CDNA) of *Solanum lycopersicum* under different irrigation levels (25%, 50%, and 100%) and treatments with *Azospirillum brasilense* and *Bacillus amyloliquefaciens*, as well as co-inoculation with both bacteria.

| Source of variation | DF | CLA | CLB | CLT | CDNA |
| --- | --- | --- | --- | --- | --- |
| Water regime (WR) | 2 | 6.4300** | 3.99535 ^NS^ | 9.1055** | 0,038383** |
| Bacteria (B) | 3 | 1.1233 ^NS^ | 0.7584 ^NS^ | 0.8807 ^NS^ | 0,008622^NS^ |
| WR x B | 6 | 1.5150 ^NS^ | 0.7982 ^NS^ | 1.7026 ^NS^ | 0,004160^NS^ |

*Significant at 5%; **Significant at 1%; and NS Not significant.

**Table S6** – Summary of the analysis of variance with degrees of freedom (DF) and mean squares for the variables: Flavonoids and Total Phenolic Compounds (Phenols) of *Solanum lycopersicum* under different irrigation levels (25%, 50%, and 100%) and treatments with *Azospirillum brasilense* and *Bacillus amyloliquefaciens*, as well as co-inoculation with both bacteria.

| Source of variation | DF | Flavonoids | Phenols |
| --- | --- | --- | --- |
| Water regime (WR) | 2 | 51.023* | 0.557^NS^ |
| Bacteria (B) | 3 | 4.002^NS^ | 2.846^NS^ |
| WR x B | 6 | 38.411** | 3.323** |

*Significant at 5%; **Significant at 1%; and NS Not significant.
