# Supplementary material for: Inoculation With Azospirillum brasilense and Bacillus amyloliquefaciens Enhances Tomato Resilience to Severe Water Deficit: A Comprehensive Morpho‐Physiological and Biochemical Analysis
Source: Environ Microbiol Rep. 2026 Apr 28;18(3):e70316. doi: 10.1111/1758-2229.70316 (PMC13124666; doi:10.1111/1758-2229.70316)
Supplement: Supplementary file 2 — Figure S1: Experimental design of potted tomato plants, distributed by blocks (5) and treatments (irrigation levels × bacteria). L1: line with 25% blade replacement; L2: line with 50% blade replacement; L3: line with 100% blade replacement; T1: control treatment without bacteria; T2: treatment with Azospirillum brasiliense; T3: treatment with Bacillus amyloliquefaciens ; and T4: treatment with a combination of Azospirillum brasiliense and Bacillus amyloliquefaciens ; P1: Plant 1 and P2: Plant 2. Figure S2: Temporal variation of temperature (°C) and relative air humidity (%) monitored in the experimental environment during the study period. Figure S3: Fluorescence histograms by flow cytometry after staining with propidium iodide. (A) Histogram for treatment with 25% slide replacement; (B) histogram for treatment with 50% slide replacement and (C) histogram for treatment with 100% slide replacement. The x‐axis represents the fluorescence intensity (reflecting the amount of stained DNA in each nucleus), while the y‐axis shows the cell count (frequency of occurrence). [file EMI4-18-e70316-s001.docx]

**SUPPLEMENTARY MATERIAL – FIGURES**


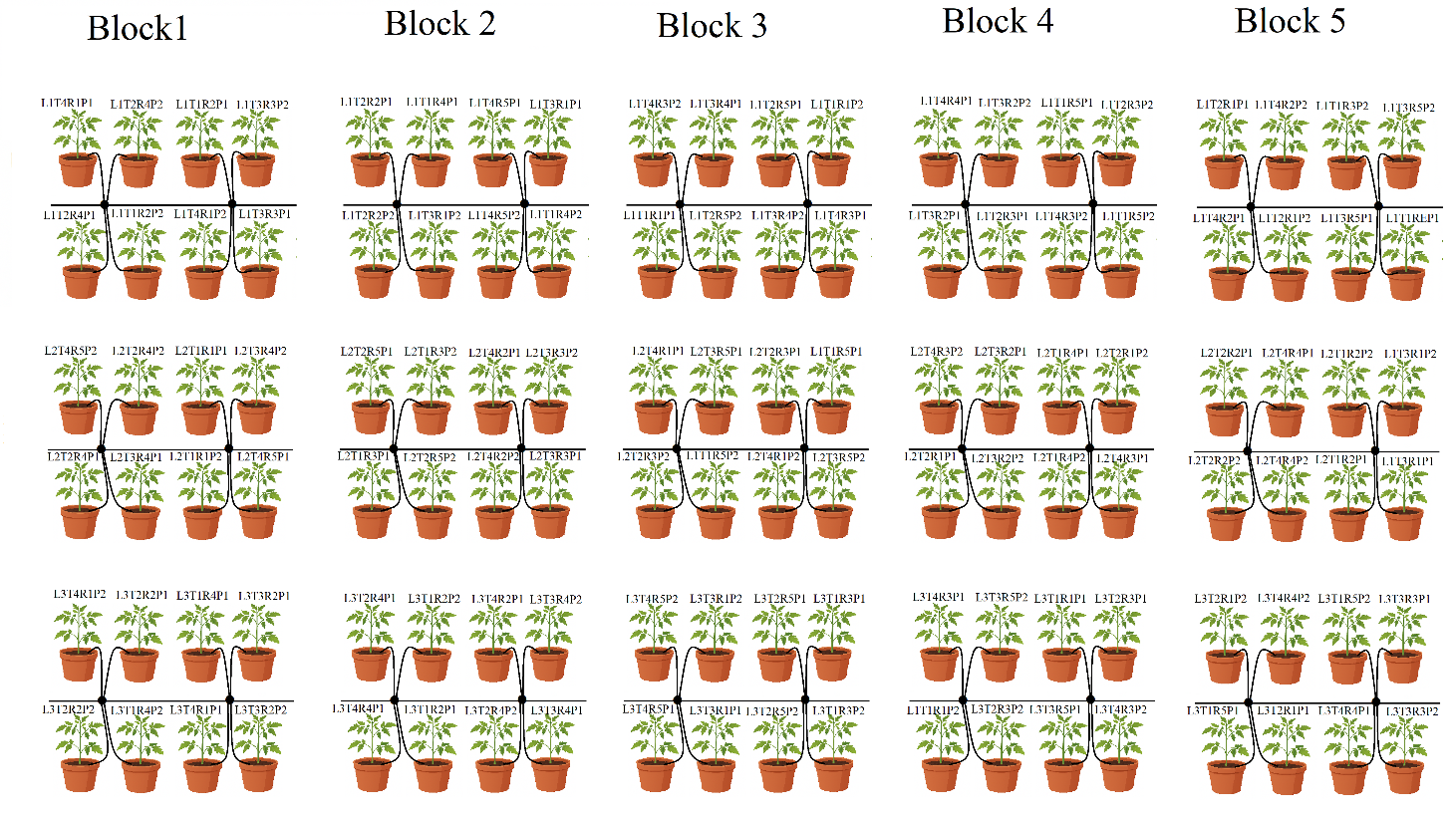


**Fig S1**: Experimental design of potted tomato plants, distributed by blocks (5) and treatments (irrigation levels x bacteria). Legend: L1: line with 25% blade replacement; L2: line with 50% blade replacement; L3: line with 100% blade replacement; T1: control treatment without bacteria; T2: treatment with *Azospirillum brasiliense*; T3: treatment with *Bacillus amyloliquefaciens*; and T4: treatment with a combination of *Azospirillum brasiliense* and *Bacillus amyloliquefaciens*; P1: Plant 1 and P2: Plant 2.


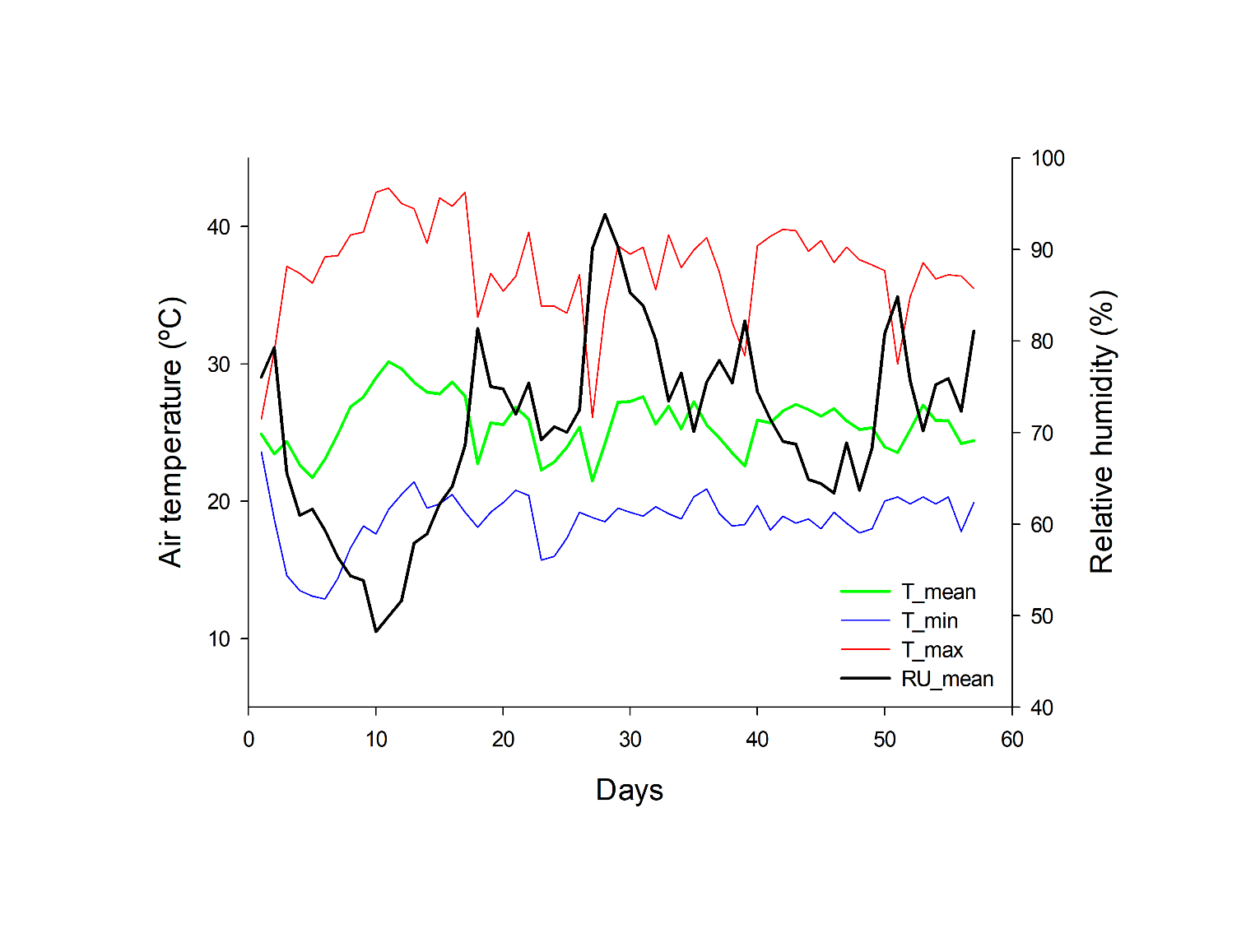


**Figure S2:**  Temporal variation of temperature (°C) and relative air humidity (%) monitored in the experimental environment during the study period.


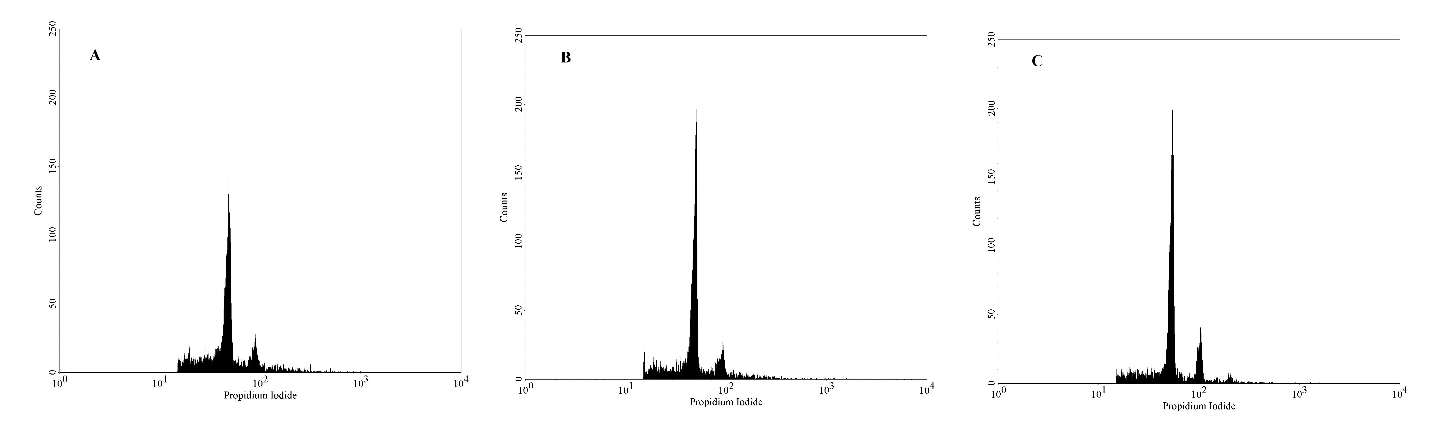
**Figure S3**: Fluorescence histograms by flow cytometry after staining with propidium iodide. In (A) histogram for treatment with 25% slide replacement; (B) histogram for treatment with 50% slide replacement; (C) histogram for treatment with 100% slide replacement. Legend: The x-axis represents the fluorescence intensity (reflecting the amount of stained DNA in each nucleus), while the y-axis shows the cell count (frequency of occurrence).
